# Shining a light on viral detection: a comparative study of electrochemical and electrochemiluminescence techniques for human cytomegalovirus

**DOI:** 10.1007/s00216-026-06410-8

**Published:** 2026-03-05

**Authors:** Aneta Fried, Karolina Itterheimova, Ludmila Moranova, Martin Bartosik

**Affiliations:** 1 Research Centre for Applied Molecular Oncology, Masaryk Memorial Cancer Institute, Zluty Kopec 7, 656 53 Brno, Czech Republic; 2 National Centre for Biomolecular Research, Faculty of Science, Masaryk University, Kamenice 5, 625 00 Brno, Czech Republic

**Keywords:** Human cytomegalovirus, Electrochemiluminescence, Electrochemistry, Cancer biomarker, Bioassay

## Abstract

Human cytomegalovirus (hCMV) is a widespread herpesvirus proposed to exert oncomodulatory effects on several cancers by influencing cell proliferation, inflammation, and immune evasion. hCMV DNA and proteins have been detected in tumors, such as glioblastoma and colorectal cancer, suggesting a possible role in tumor biology. However, detection rates in clinical samples vary dramatically from 0 to 100%, reflecting both biological heterogeneity and methodological differences. Standard detection methods such as immunohistochemistry, qPCR, and sequencing provide good sensitivity, but each has limitations, including inconsistent antibody performance, costly instrumentation, and limited suitability for routine screening in point-of-care settings. Electrochemical (EC) and electrogenerated chemiluminescence (ECL) bioassays and biosensors offer attractive alternatives, providing rapid, simple, and low-sample-consumption assays that are well suited for point-of-care testing. These platforms combine high analytical sensitivity with minimal instrumentation requirements, short turnaround times, and compatibility with amplification strategies, making them promising tools for decentralized viral diagnostics. In this study, we optimized and compared EC and ECL bioassays coupled with various amplification strategies, including PCR, recombinase polymerase amplification (RPA), and loop-mediated isothermal amplification (LAMP), for hCMV detection in real samples. Amperometric EC readout combined with PCR or RPA showed high specificity, reliably distinguishing hCMV-infected from uninfected cells, and demonstrating its potential for use in tumor tissue analysis. LAMP offers excellent sensitivity but suffers from nonspecific amplification, whereas the ECL assay failed to detect viral DNA under our conditions. These results highlight the relative strengths and limitations of these platforms and indicate their suitability for future hCMV diagnostics.

**Graphical Abstract:**

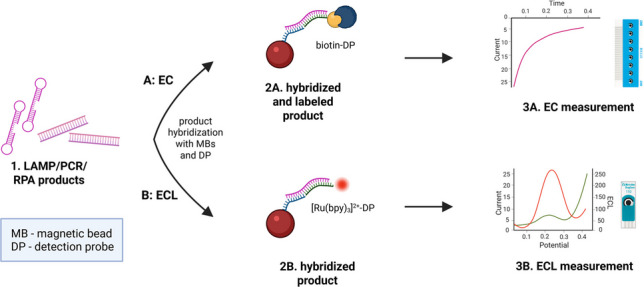

**Supplementary Information:**

The online version contains supplementary material available at 10.1007/s00216-026-06410-8.

## Introduction

Human cytomegalovirus (hCMV) is a double-stranded DNA (dsDNA) virus of the Herpesviridae family, which is characterized by its ability to persist and reactivate in the host organism. The prevalence of hCMV is markedly high, reaching approximately 70% in industrialized countries and up to 100% in developing countries [1]. In healthy populations, hCMV infection is usually asymptomatic; however, in immunocompromised individuals, primary infection or reactivation can lead to severe clinical manifestations [2].

Up to 12% of all cancers are linked to viral infections [3]. Although hCMV is not traditionally classified as an oncovirus, increasing evidence suggests that it is involved in cancer development. The oncomodulatory properties of hCMV have been known for a long time, and recent studies have further emphasized their association with various malignancies, including glioblastoma and breast cancer [4–6].

The detection of hCMV in cancer patients remains a highly debated and actively studied area of research. The prevalence of hCMV in blood and cancer tissue samples varies widely, depending on the biological material used, detection methods, performance, and epidemiological differences. Therefore, the positive findings range widely from 0 to 100% [7]. Diagnostic assays are generally divided into direct detection assays, which identify viral components like DNA, RNA, or proteins, and indirect approaches, which assess the host immune response by measuring specific antibodies such as IgG, IgM, or IgA. Antibody-based assays reflect prior exposure or immune status rather than the current presence of hCMV, which limits their usefulness in immunocompromised or cancer patients. Therefore, in such cases, direct detection methods are preferred, and testing is typically performed on tumor tissues using techniques such as immunohistochemistry and quantitative PCR (qPCR).

Immunohistochemistry is a routine diagnostic method for diseases with organ involvement, usually focusing on the CCH2/DDG9 antibody against the hCMV DNA-binding pp52 protein antigen. However, this protein is only expressed during active viral replication and is thus not ideal for detection in tumor tissues [8]. Conversely, pp65, a major tegument protein of hCMV, plays a pivotal role in modulating the host immune response and has been identified in a significant percentage of various tumor types, including those of the central nervous system, breast, prostate, and other cancers [9–12].

qPCR is the basis of most commercial kits. Although qPCR is widely used, it requires costly instrumentation and is relatively time-consuming. Sequencing methods such as next-generation sequencing (NGS) and third-generation sequencing are available, but they are expensive, and to date, no studies have reported successful hCMV detection in glioblastoma samples using these techniques [7].

Isothermal amplification techniques (IATs) are attractive alternatives to PCR, as they operate at a constant temperature and eliminate the need for a thermocycler. The best-known IAT is loop-mediated amplification (LAMP), a reaction based on Bst polymerase, using 4–6 primers at a constant temperature of 60–65 °C. Recombinase polymerase amplification (RPA) is a more recent method that was first published by Piepenburg et al. in 2006 [13]. True to its name, RPA is driven by recombinase and polymerase, which are essential alongside a single-stranded DNA-binding protein. RPA works best at lower temperatures (37–42 °C).

These IATs provide rapid and sensitive detection and are particularly well suited for integration with novel analytical tools based on EC or ECL readouts, utilizing either single electrodes or electrode chips and arrays. Bioassays and biosensors that employ EC analysis benefit from cost-effective, simple, and miniaturized instrumentation. This setup permits parallel measurements of electrode chips, enabling rapid and highly sensitive determinations that are ideal for decentralized medicine and point-of-care (POC) diagnostics. EC methods are increasingly being applied for the analysis of tumor biomarkers based on nucleic acids [14–17]. For instance, our research group has successfully developed various EC-based assays combined with IATs for the detection of oncoviral HPV DNA [18–22] or HPV RNA [23], DNA point mutations [24–26], and non-coding RNAs [27, 28].

In contrast to EC, ECL is an optical detection technique that is also performed on electrode surfaces. ECL operates by generating excited-state species via electrochemical reactions at the electrode, which subsequently undergo energy transfer processes, resulting in light emission captured by an ECL detector. The primary advantages of ECL are its high sensitivity, low background noise, inherent multiplexing capabilities, rapid detection times, and potential for implementation in fully automated devices in clinical laboratories [29–32].

However, to the best of our knowledge, these sensitive EC-based techniques have only been applied to the analysis of hCMV in real clinical samples in one previous study [33]. In this methodological study, we systematically optimized and compared EC and ECL endpoint detection platforms using multiple amplification techniques, including PCR, RPA, and LAMP, to evaluate their performance in detecting hCMV in real samples and provide a critical assessment of their strengths and limitations. Our results demonstrate that a bioassay based on amperometric detection combined with PCR or RPA is highly specific and effectively distinguishes between cell lines infected with hCMV strains and uninfected controls, highlighting its potential for detecting hCMV in clinical tumor tissue samples. We also found that the LAMP reaction is extremely sensitive, but prone to nonspecific amplification. In contrast, the ECL-based bioassay was unable to detect viral DNA in this study despite being widely recognized for its high sensitivity.

## Material and methods

The list of oligonucleotide sequences, different screen-printed electrodes, and cyclic voltammetry parameters is described in detail in the Supplementary Information.

### Material

Master mix for LAMP reaction (Saphir Bst Turbo GreenMaster) was from Jena Bioscience (Germany), master mix for PCR reaction (PPP Master Mix) was from TopBio (Czech Republic), TwistAmp® Basic kit used for RPA reaction was from TwistDx Ltd. (UK), Sera-Mag carboxylate-modified magnetic beads were from Cytiva (USA), PolyHRP-Streptavidin (N200), casein blocking buffer (CBB, sold as Blocker Casein in PBS), and MeltDoctor HRM MasterMix were from Thermo Fisher Scientific (USA), GeneProof® Cytomegalovirus (CMV) PCR Kit was from GeneProof (Czech Republic), 2-(N-morpholino)ethanesulfonic acid (MES), potassium chloride, EDTA, streptavidin-peroxidase monomer from *Streptomyces avidinii* (S5512), ultrasensitive streptavidin-peroxidase polymer (S2438), hydroquinone (HQ), tris(2,2′-bipyridyl)ruthenium(II)chloride hexahydrate ([Ru(bpy)_3_]^2+^), tripropylamine (TPA), and Tween® 20 were all from Sigma-Aldrich (USA), boric acid, sodium chloride, sodium phosphate dibasic dodecahydrate, and potassium phosphate monobasic were from Lachner (Czech Republic), sodium phosphate monobasic dehydrate was from Penta (Czech Republic), Tris-HCl was from Affymetrix (USA), agarose for gel electrophoresis, 1-ethyl-3-(3-dimethylaminopropyl)carbodiimide (EDC) and Tris base were from The Carl Roth GmbH (Germany) and GelRed nucleic acid gel stain was from Biotium (USA). All other chemicals were of analytical grade and all solutions were prepared using deionized water.

All oligonucleotides were synthesized by Metabion (Germany), except for the detection probe (DP) for ECL modified with [Ru(bpy)_3_]^2+^, which was synthesized by DIANA Biotechnologies (Czech Republic). LAMP primers were designed with PrimerExplorer V.5 software (https://primerexplorer.eiken.co.jp/lampv5e/index.html). The sequences of all oligonucleotides are listed in Table S1 (Supplementary Information). The modified magnetic beads were quantified using an Eppendorf 6131 Biophotometer Spectrometer (Germany). EC measurements were performed using a multipotentiostat/galvanostat µStat 8000 (Metrohm DropSens, Spain) connected to an electrochemical array of eight cells, each in a three-electrode setup (Metrohm DropSens, DRP-8×110) controlled by DropView 8400 software. ECL readings were obtained on single screen-printed carbon electrodes (SPCE) (Metrohm DropSens, DRP-110) using µStatECL potentiostat combined with dedicated spectrometer and photodiode detection cell (Metrohm DropSens, Spain) and were controlled by DropView SPELEC software. A horizontal gel electrophoresis system (Sub Cell GT Cell and Mini-Sub Cell GT Cell) was purchased from Bio-Rad (USA). The following buffers were used: MES buffer for magnetic beads modification (25 mM MES buffer, pH 5.0); TBE buffer for gel electrophoresis (0.1 M Tris base, 0.1 M boric acid, and 2 µM EDTA, pH 8.0); washing buffer (WB) for magnetic beads (1 M NaCl, 0.5 mM EDTA, and 5 mM Tris-HCl, pH 8.5); phosphate buffer (PB, 0.1 M sodium phosphate buffer, pH 6.0); 1× PBS (0.14 M NaCl, 2.7 mM KCl, 1.5 mM KH_2_PO_4_, 6.4 mM Na_2_HPO_4_); and homogenization buffer (5 mM Tris-HCl, pH 8.5).

### Cell lines and DNA extraction

The cervical cancer cell lines HeLa and SiHa, colorectal adenocarcinoma cell line SW620, human melanoma cell line A375, and lung fibroblast cell line MRC-5 were cultivated in Dulbecco’s Modified Eagle’s Medium (DMEM) (Merck, USA) containing 1% pyruvate, 1% penicillin-streptomycin (Biosera, France), and 10% fetal bovine serum (FBS, Gibco, USA). MRC-5 cells were infected with either hCMV laboratory strain AD169 (ATCC, USA) or clinical strain Merlin (ATCC, USA). All cells were cultivated at 37 °C in a humidified atmosphere containing 5% CO_2_. DNA extraction was performed using Tissue DNA Preparation - Column Kit (Jena Bioscience, Germany), according to the manufacturer’s instructions.

### LAMP reaction

The LAMP reaction mix contained 2× Saphir LAMP Turbo GreenMaster, diluted 1× in the final reaction. Outer primers F3/B3 were added at a final concentration of 0.2 µM, inner primers FIP/BIP at 1.6 µM, and loop primers LF/LB at 0.4 µM. A total of 100 ng of template DNA was added to each reaction mixture. No-template controls (NTCs) were included for each run. The reactions were carried out at 62 °C for 30 min. LAMP products were visualized by agarose electrophoresis using a 1.5% gel prepared in 0.5× TBE buffer and stained with GelRed (1:10,000 dilution).

### RPA reaction

RPA reactions were performed using the TwistAmp® Basic kit. Forward and reverse primers were added at a final concentration of 1.6 µM. After preparing the master mix, the lyophilized enzyme pellet was reconstituted, and the reaction volume was evenly divided into three tubes of 15 µL, with the volume of all component ratios adjusted accordingly. A total of 100 ng of template DNA was added to each reaction. NTCs were included in each run. The mixture was then incubated in a thermoblock at 39 °C for 20 min. RPA products were visualized by agarose electrophoresis using a 1.5% gel prepared in 0.5× TBE buffer and stained with GelRed (1:10,000 dilution).

### PCR reaction

The PCR reaction mix contained 2× PPP Master Mix diluted to 1× in the final reaction. Forward and reverse primers were added at final concentration of 0.67 µM. A total of 100 ng of template DNA was added to each reaction. NTCs were included in each run. The PCR reaction was run using the following program: 94 °C for 3 min, 40 cycles of 94 °C for 15 s, 65 °C for 15 s, and 72 °C for 30 s, followed by a 7-min final extension. PCR products were visualized by agarose electrophoresis using a 1.5% gel prepared in 0.5× TBE buffer and stained with GelRed (1:10,000 dilution).

### Magnetic beads preparation

Magnetic beads (MBs) were modified with amino-labeled capture probes as follows: 20 µL of MBs were washed three times with 190 µL of 25 mM MES buffer (pH 5.0), with each washing step followed by incubation for 10 min at room temperature with rotation. Then, 20 µL of 100 µM capture probe (2 nmol) was added to the MBs and incubated for 30 min at RT with rotation. Without washing, 30 µL of 100 mg/mL EDC (dissolved in MES buffer directly before use) was added to the MBs solution and incubated overnight at 4 °C on a rotator. The modified MBs were washed three times with 190 µL PBS, resuspended in 200 µL PBS with 0.02% sodium azide, and stored at 4 °C. The amount of MBs for further hybridization was determined by measuring the optical density at 600 nm (OD600) to ensure that the same amount of MBs was used across all batches.

### Hybridization on magnetic beads for EC measurement

After DNA amplification, samples were denatured by incubation at 95 °C for 10 min. Modified magnetic beads were washed twice with 100 µL of WB and twice with 100 µL of CBB and incubated with 3.5 µL of the denatured LAMP, RPA, or PCR product, 0.6 M NaCl, and 0.5 µM biotin-DP at 40 °C for 15 min. The MBs were then washed three times with CBB and incubated in 50 µL of streptavidin-peroxidase polymer (SPP) (dilution 1:1000 in CBB) at room temperature for 15 min. Finally, the MBs were washed three times with PB and resuspended in 10 µL PB.

### EC measurement

Resuspended MBs with hybridized DNA were transferred onto the working electrode with magnetic support placed underneath. The electrode was then covered with 50 µL of substrate solution containing 50 mM H_2_O_2_ and 10 mM hydroquinone. Amperometry was performed at −0.3 V for 60 s to monitor the reduction of enzymatically oxidized HQ.

### Hybridization on magnetic beads for ECL measurement

The amplified products were denatured, and the MBs were washed the same way as described for the EC measurement. The MBs were then incubated with 5 µL of denatured LAMP, RPA, or PCR product, 0.6 M NaCl, and 0.5 µM [Ru(bpy)_3_]^2+^-DP at 40 °C for 15 min. Finally, the MBs were washed three times with PBS.

### ECL measurement

The MBs with hybridized DNA were resuspended in 70 µL PBS containing 40 mM TPA as a coreactant. The SPCE was placed into the detection cell, and the MB suspension was added sequentially to cover the entire electrode surface in a total volume of 60 µL. The detection cell was properly closed and connected to the µStatECL potentiostat. ECL was measured using linear sweep voltammetry (LSV) from +0.3 V to +1.8 V, using a scan rate of 0.5 V/s and E_step_ of 0.002 V. For measurements using a photodiode detection cell, a 100× amplification was used, whereas for the spectrophotometer detection cell, spectra acquired at 1.3 V were evaluated.

### HRM analysis

High-resolution melting (HRM) analysis was performed using MeltDoctor HRM MasterMix 2× (Thermo Fisher Scientific) diluted to 1×, with target oligonucleotides and detection probes added to a final concentration of 5 µM each. Samples were analyzed using a QuantStudio™ 5 Real-Time PCR System (Thermo Fisher Scientific) and QuantStudio software. The HRM cycle was conducted as follows: 95 °C for 18 s, ramping at 3 °C/s; 30 °C for 90 s, ramping at 2 °C/s; 95 °C for 6 s, ramping at 0.037 °C/s; followed by 30 °C for 9 s, ramping at 2 °C/s.

### Quantitative PCR (qPCR)

A commercial kit for hCMV analysis (Cytomegalovirus PCR Kit from GeneProof) was used according to the manufacturer’s instructions with a QuantStudio™ 5 Real-Time PCR System (Thermo Fisher Scientific) connected to QuantStudio software. NTCs were included in each run. The thermal cycling protocol consisted of an initial step at 37 °C for 2 min, followed by 95 °C for 10 min, and 45 cycles of 95 °C for 5 s, 60 °C for 40 s, and 72 °C for 20 s. 6-Carboxyfluorescein (FAM) was used as a reporter dye.

### Matrix spiking with synthetic targets

The cell pellet was thawed and resuspended in 500 µL of homogenization buffer, followed by incubation at 98 °C for 5 min. For hybridization on MBs, 9 µL of the resulting lysate and 2.5 µL of synthetic target were combined with the hybridization mix, 0.6 M NaCl, and 0.5 µM biotin-DP. The final reaction volume (25 µL) was incubated at 40 °C for 15 min. Further hybridization and EC measurements were performed as described above.

### Data analysis

hCMV DNA quantification was performed using the GeneProof® Cytomegalovirus (CMV) PCR Kit, which includes four quantitative calibrators ranging from 10^1^ to 10^4^ IU/µL. The viral DNA concentrations of the hCMV laboratory strains AD169 and Merlin were determined from the calibration curve constructed by plotting the cycle threshold (Ct) values against the logarithm of the calibrator concentrations.

All statistical analyses were performed using GraphPad Prism software (version 7.03) and calculated from triplicate measurements. The limit of detection (LOD) was determined according to the 3σ criterion and calculated as three times the standard deviation of the blank signal divided by the slope of the linear part of the concentration dependence.

The ECL measurements were quantified as the peak area under the emission curve, exhibiting a peak maximum at ~ 1.3 V.

## Results and discussion

### Assay principles

Several conditions were tested and optimized to determine the most effective detection protocols. These included testing different types of biotin labels or streptavidin-peroxidase conjugates, evaluating various times and temperatures of individual steps, and adjusting multiple parameters of the EC and ECL techniques (Table S2). Based on the literature, the *US28* gene was selected as a key biomarker involved in oncomodulation [34–36]. Primers for LAMP, PCR, and RPA were designed according to specific guidelines for each amplification method [37]. All primer sets targeted the same region of the *US28* gene to ensure consistency across detection techniques and were homologous to both tested hCMV strains (AD169 and Merlin). During testing, the RPA primers (hCMV_US28_RPA_F/R) produced stronger amplification signals than the F3/B3 RPA primer pair; however, the NTC reaction also generated high nonspecific EC signals. Even after testing several blocking conditions, the nonspecific signals could not be eliminated. As a result, the F3/B3 primer sequences were adopted for all three amplification methods, used alone in RPA and PCR, and together with FIP/BIP and LF/LB primers in the LAMP assay. This ensured that all amplified products could hybridize with a single capture probe, thereby reducing the number of probes necessary for analysis.

**Fig. 1:**
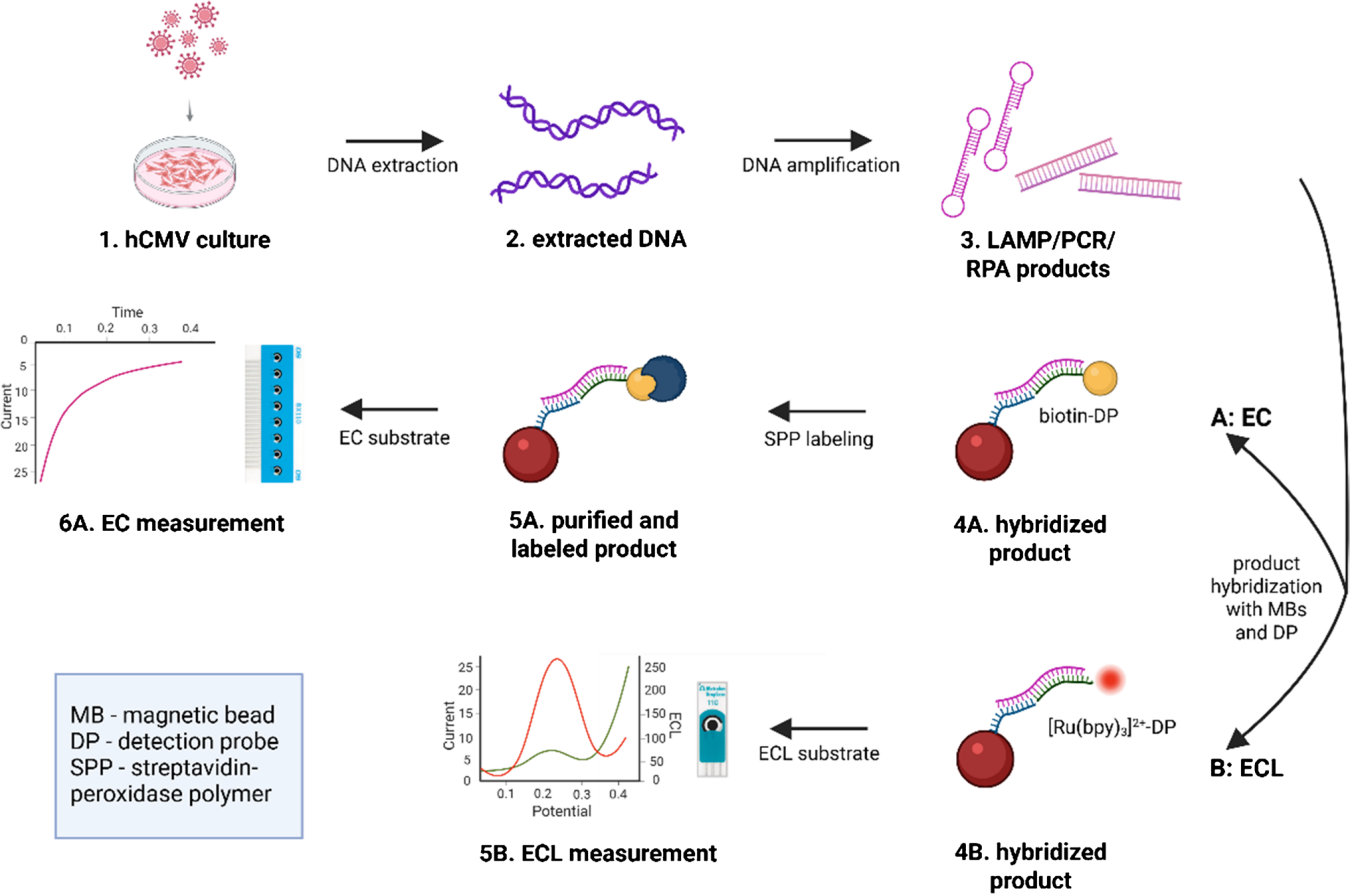
MRC-5 cells infected with hCMV serve as the source of viral DNA (step 1). The extracted DNA (step 2) is introduced into the amplification reaction using LAMP, PCR, or RPA (step 3). The resulting products are hybridized with capture probe (CP)-modified magnetic beads (MBs) and the respective detection probe (DP): biotin-DP for the EC protocol (step 4 A) and [Ru(bpy)_3_]^2+^-DP for the ECL protocol (step 4B). In the EC protocol, hybridization is followed by incubation with streptavidin-peroxidase polymer (SPP) (step 5 A). Finally, the MB complexes are transferred onto the carbon electrode for signal measurement using either EC (step 6 A) or ECL readout (step 5B)

DNA from the hCMV-infected MRC-5 fibroblast cell line (strain AD169), containing both viral and human genomic DNA, was used as a positive control (Fig. 1(1)). Extracted DNA from the cell lines (Fig. 1(2)) was used as a template for the amplification reaction (Fig. 1(3)). The LAMP reaction was performed for 30 min at 62 °C, RPA reaction for 20 min at 39 °C, and PCR reaction for approximately 1.5 h. Amplified products (serving as targets in the subsequent hybridization step at MBs) were denatured for 10 min at 95 °C and then mixed with MBs covalently modified with capture probes (CP). Subsequently, the MBs modified with the amplification products were hybridized with labeled DP for 15 min at 40 °C (Fig. 1(4)). Two labeled DPs were tested: [Ru(bpy)₃]^2^⁺-DP for the ECL protocol (Fig. 1(4B)), where the MBs were washed after hybridization and measured using TPA as a coreactant (Fig. 1(5B)) and biotin-DP for the EC protocol (Fig. 1(4A)), in which biotin bound to SPP during a 15 min incubation at room temperature (Fig. 1(5A)). After washing, the complex was measured amperometrically using the HQ/H_2_O_2_ couple as a substrate for the peroxidase reaction (Fig. 1(6A)).

### Optimization of ECL assay

As described previously, the ECL workflow employed a [Ru(bpy)_3_]^2+^-labeled DP with an oligonucleotide sequence identical to that of biotin-DP used in EC measurements to ensure comparability. A series of preliminary optimization experiments was performed to establish the proper operation and sensitivity limits of the ECL measurement setup. These experiments employed the conventional [Ru(bpy)_3_]^2+^/TPA system in a homogeneous solution and were intended to validate key instrumental and EC parameters prior to transitioning to the more complex bead-based ECL format. In contrast, the ECL generation mechanism in solution differs from that in bead-based assays, where the luminophore is immobilized on insulating magnetic beads, and ECL emission is predominantly governed by TPA oxidation at the electrode surface. Hence, these solution-phase experiments served as an essential control to confirm reliable ECL performance and signal stability of the platform.

Owing to the availability of both a spectrometer and a photodiode detection cell with signal amplification capability, all optimizations were performed under both detection settings. For the photodiode cell, the measurements were compared at signal amplification levels of 1×, 10×, and 100×. Previous studies have shown that the intensity of the ECL signal is affected by scan rate [38]. Therefore, scan rates ranging from 0.05 to 0.5 V/s were tested. Based on signal intensity, reproducibility, and analysis time, a scan rate of 0.5 V/s was selected for all subsequent optimizations (Fig. S1).

To achieve the highest signals, various measurement buffers and [Ru(bpy)_3_]^2+^/TPA concentrations were tested according to the literature recommendations [39–41], along with different screen-printed electrode materials. Common electrode materials utilized for ECL are carbon and gold; however, nanomaterial-modified electrodes are often employed because of their superior detection properties. The influence of surfactants such as Tween 20 has also been studied, demonstrating that their presence increases the hydrophobicity of noble metal electrodes, thereby allowing easier TPA oxidation followed by luminophore excitation [40].

The buffer performance was evaluated using 1 nM and 50 nM [Ru(bpy)_3_]^2+^; in both cases, 40 mM TPA consistently yielded the highest signals and therefore was used for all subsequent experiments (Fig. S2). The unmodified carbon electrode demonstrated superior ECL signals among all tested electrode materials (Fig. S3). Both optimizations were performed on a photodiode using a 100× amplification setup.

**Fig. 2:**
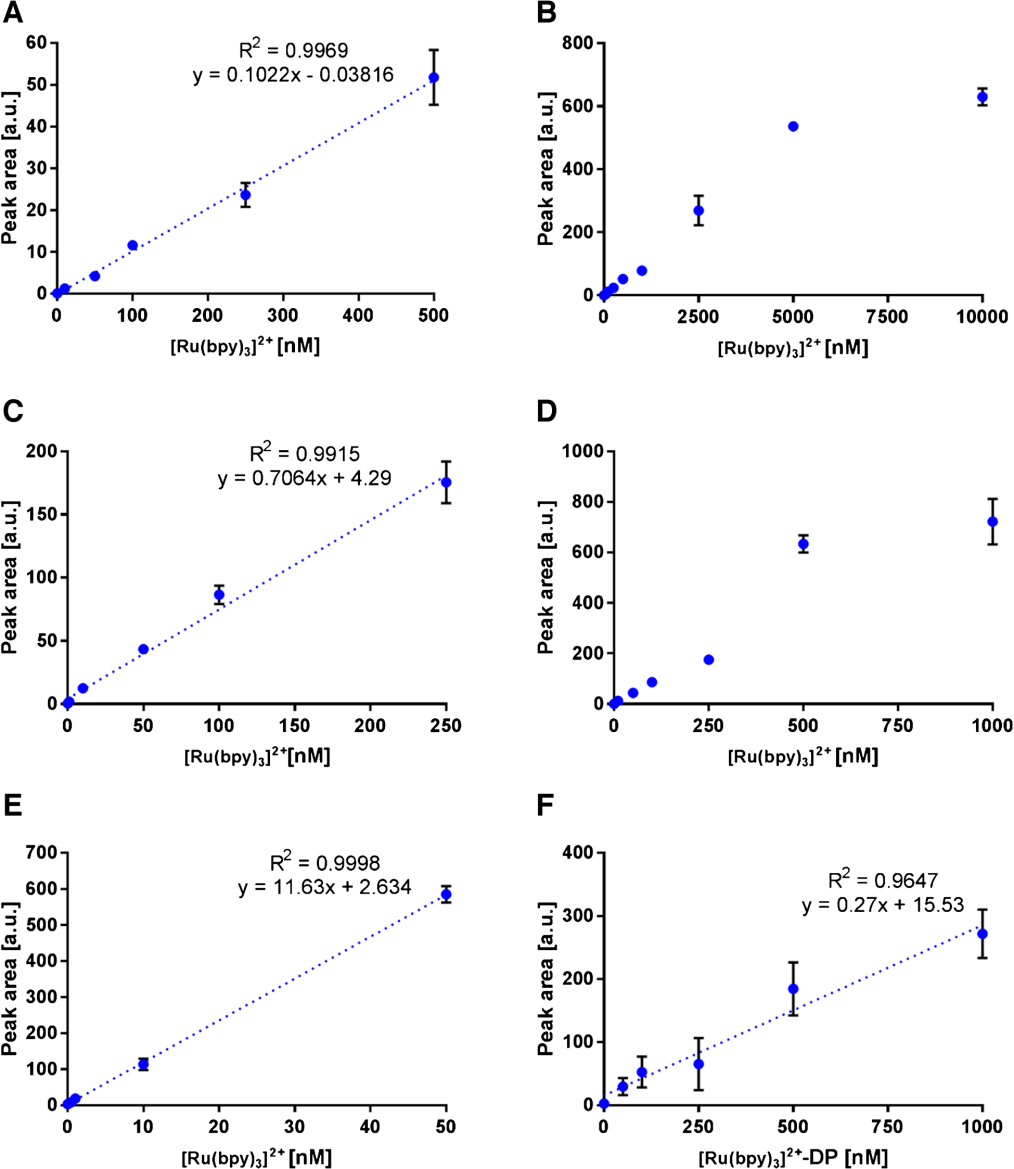
ECL measurements of different [Ru(bpy)_3_]^2+^ concentrations. **A**, **B** ECL of [Ru(bpy)_3_]^2+^ measured using a photodiode without amplification (1×) in the range of 0.1–10,000 nM. **C**, **D** ECL of [Ru(bpy)_3_]^2+^ measured using a photodiode with 10× amplification in the range of 0.1–1000 nM. **E** ECL of [Ru(bpy)_3_]^2+^ measured on a photodiode with 100× amplification in the range of 0.1–50 nM. **F** ECL of [Ru(bpy)_3_]^2+^-DP measured on a photodiode with 100× amplification in the range of 1–1000 nM. Concentration of TPA was 40 mM

To achieve the best sensing ability, several aspects of ECL measurements were studied together with the selection of the ECL interface. Using the optimized scan rate and buffer composition, the linear range and LOD of [Ru(bpy)_3_]^2+^ were determined for all four measurement setups. Photodiode with 1× amplification (Fig. 2A, B) achieved a LOD of 1.75 nM with a linear range of 1 to 500 nM (R^2^ = 0.9969), photodiode with 10× amplification (Fig. 2C, D) achieved LOD of 0.21 nM with linear range of 0.5 to 250 nM (R^2^ = 0.9915), photodiode with 100× amplification (Fig. 2E) achieved LOD of 0.03 nM with linear range of 0.1 to 50 nM (R^2^ = 0.9998), and finally, detection on spectrometer cell (Fig. S4) achieved LOD of 0.11 µM with linear range of 0.25 to 5 µM (R^2^ = 0.9911). As the biomarker levels were expected to be very low, the photodiode setup with 100× signal amplification was selected to achieve the lowest possible LOD and thus ensure the most sensitive ECL measurements. The narrow linear range observed at 100× photodiode amplification results from amplification limitations, where high [Ru(bpy)_3_]^2+^ concentrations produce an ECL signal that saturates the detector. Under the same conditions, the LOD (8.88 nM) and linear range (50 nM to 1 µM) of [Ru(bpy)_3_]^2+^-DP on 100× photodiode were determined (Fig. 2F).

Fig. S5 presents a representative ECL–LSV curve recorded simultaneously under our experimental conditions using 50 nM [Ru(bpy)_3_]^2+^ and 40 mM TPA in solution. In addition, in Fig. S6 we performed cyclic voltammetry (CV), instead of LSV, as an alternative EC technique commonly employed for ECL measurements. As shown in Fig. S6B, the ECL response obtained by CV exhibits essentially identical peak characteristics to those observed using LSV.

### Optimization of EC assay

**Fig. 3:**
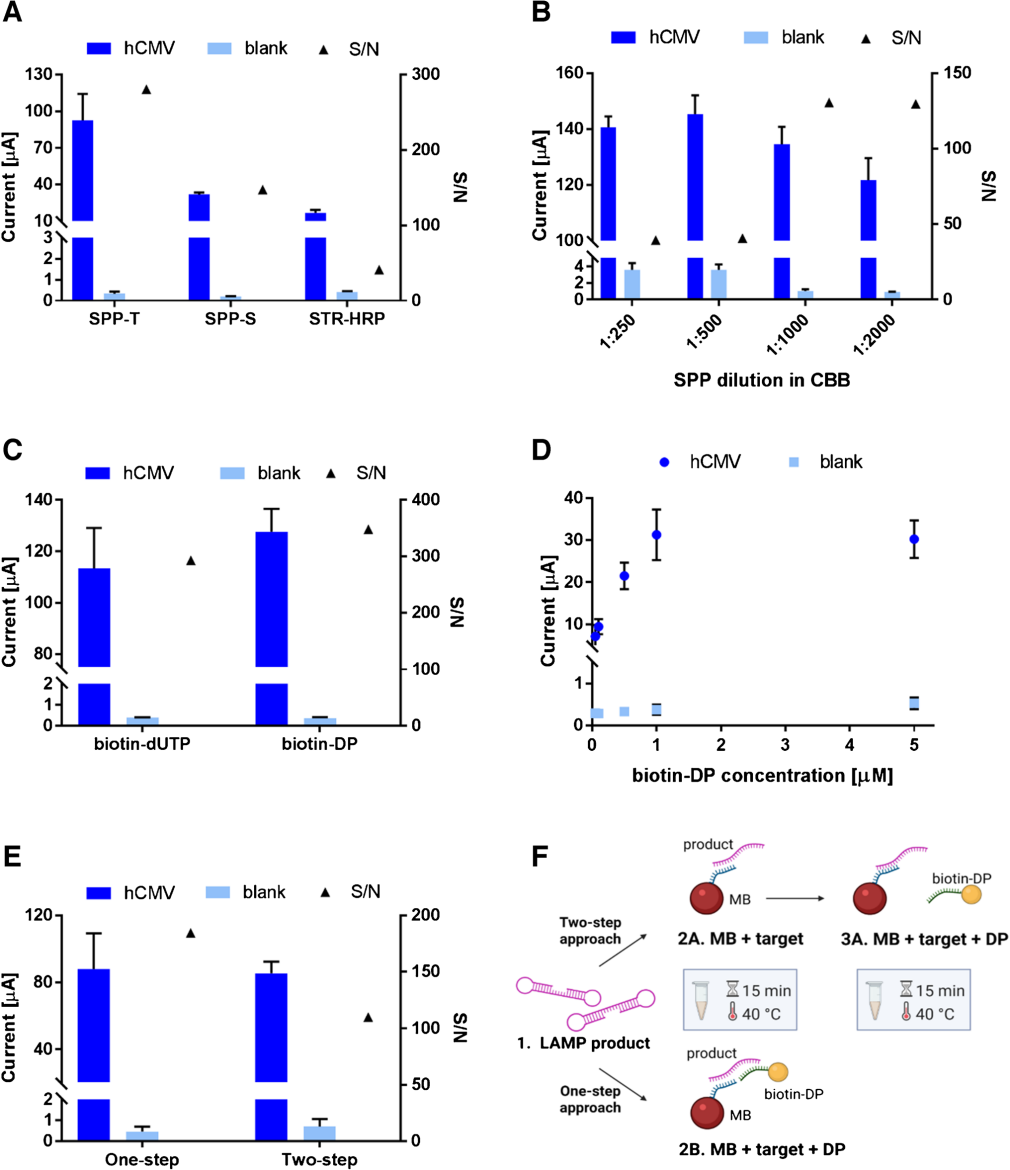
Optimization of the assay with EC endpoint detection. **A** Comparison of HRP types—streptavidin-peroxidase polymer (SPP) from two suppliers and monomer (STR-HRP). **B** SPP-T dilution optimization. **C** Determination of the difference in the use of biotin-dUTP incorporated into LAMP amplicons and post-amplification labeling by biotin-DP. **D** Concentration dependence of biotin-DP in the reaction mixture. **E** Comparison of hybridization of LAMP product on magnetic beads and biotin-DP labeling as combined (One-step) and separated (Two-step) procedures. **F** Schematic representation of two alternative strategies for signal amplification. In the Two-step approach, the amplified product (step 1) is first hybridized with MBs (step 2 A), followed by separate incubation with DP (step 3 A). In the One-step approach, all the components are incubated simultaneously in a single reaction mixture (step 2B). Both protocols were performed for 15 min at 40 °C

Multiple optimization steps were performed to identify the most suitable conditions for reliable comparison between the EC and ECL setups (Table S2). Most parameters of the EC workflow were optimized using the LAMP reaction, based on previous experience [24]. Among the conditions tested was the use of horseradish peroxidase conjugated to streptavidin. Based on our previous studies [24, 25, 27], we tested three variants of horseradish peroxidase conjugate at a 1:1000 dilution (Fig. 3A): horseradish peroxidase monomer (STR-HRP) and horseradish peroxidase polymer from two suppliers, that is SPP from Thermo Fisher Scientific (SPP-T) and SPP from Sigma-Aldrich (SPP-S). For all further experiments, we used SPP-T because it provided the highest signal and thus the best sensitivity. The selected SPP was tested at dilutions ranging from 1:250 to 1:2000 (Fig. 3B). Taking into account not only the signal intensity and signal-to-noise ratio, but also the relative standard deviations (1:1000 RSD = 3.76; 1:2000 RSD = 5.24), a dilution of 1:1000 was chosen for further experiments.

In previous EC studies, biotin-dUTP was incorporated into the LAMP reaction instead of DPs [24, 25]. To ensure methodological consistency with the current ECL protocol employing [Ru(bpy)_3_]^2+^-labeled DP, biotin-labeled DP was used for EC measurements, and two biotin-labeling strategies were compared (Fig. 3C), revealing similar performance under these conditions. The biotin-DP sequence was selected after evaluating four different DP designs within the amplified region (the tested sequences are listed in Table S1). Owing to the additional hybridization step, sequence-specific biotin-DPs provide higher target selectivity than biotin-dUTP. Therefore, biotin-DP was selected for all subsequent EC detection experiments.

Moreover, because SPP-T generated substantially stronger signals, distinguishing subtle effects during optimization (e.g., DP concentration, Fig. 3D) became difficult. Therefore, for these optimization experiments, we used a lower-signal SPP-S. Based on signals of measured concentration of biotin-DP (Fig. 3D), 0.5 µM concentration was selected. Higher concentrations showed an increase of signal as well as higher SD and hook effect. For the biotin-DP approach, we assessed whether the signal intensity could be enhanced using one incubation step combining all components in a single mixture (MB + target + DP) or by performing two separate incubation steps (MB + target, followed by DP). These strategies are illustrated in Fig. 3E and F as the One-step and Two-step approaches. Considering both efficiency and performance, the One-step protocol, in which MB, the amplified product, and DP were incubated simultaneously, was selected. This method requires only two 15 min incubations and provides high signal intensity with a favorable signal-to-noise ratio and very good reproducibility of < 6% RSD from 12 independent measurements (Table S3). The fluctuation in the signal among various measurements could have been caused by variability in the efficiency of LAMP amplification.

**Fig. 4:**
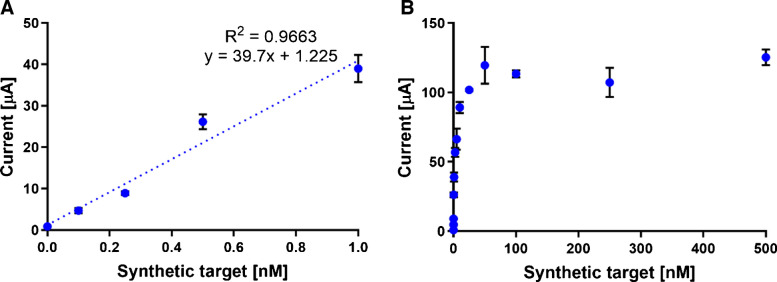
EC measurements. **A** Linear region of the concentration dependence of the synthetic target from 0 to 1 nM. **B** Concentration dependence of the synthetic target from 0 to 500 nM

For the following experiment, a mixture of synthetic target and its complementary sequence was used to create dsDNA oligonucleotides to simulate dsDNA amplicons. The synthetic target covered a partial sequence of amplicons, including hybridization regions for CP and DP. To determine the concentration range, the synthetic target was measured in the range from 0.1 to 500 nM (Fig. 4). The linear range was determined from 0.1 to 1 nM (Fig. 4A), followed by a nonlinear region and saturation above 50 nM owing to the hook effect (Fig. 4B). The LOD of the synthetic target was determined to be 0.01 nM. The measured values are presented in Table S4.

**Fig. 5:**
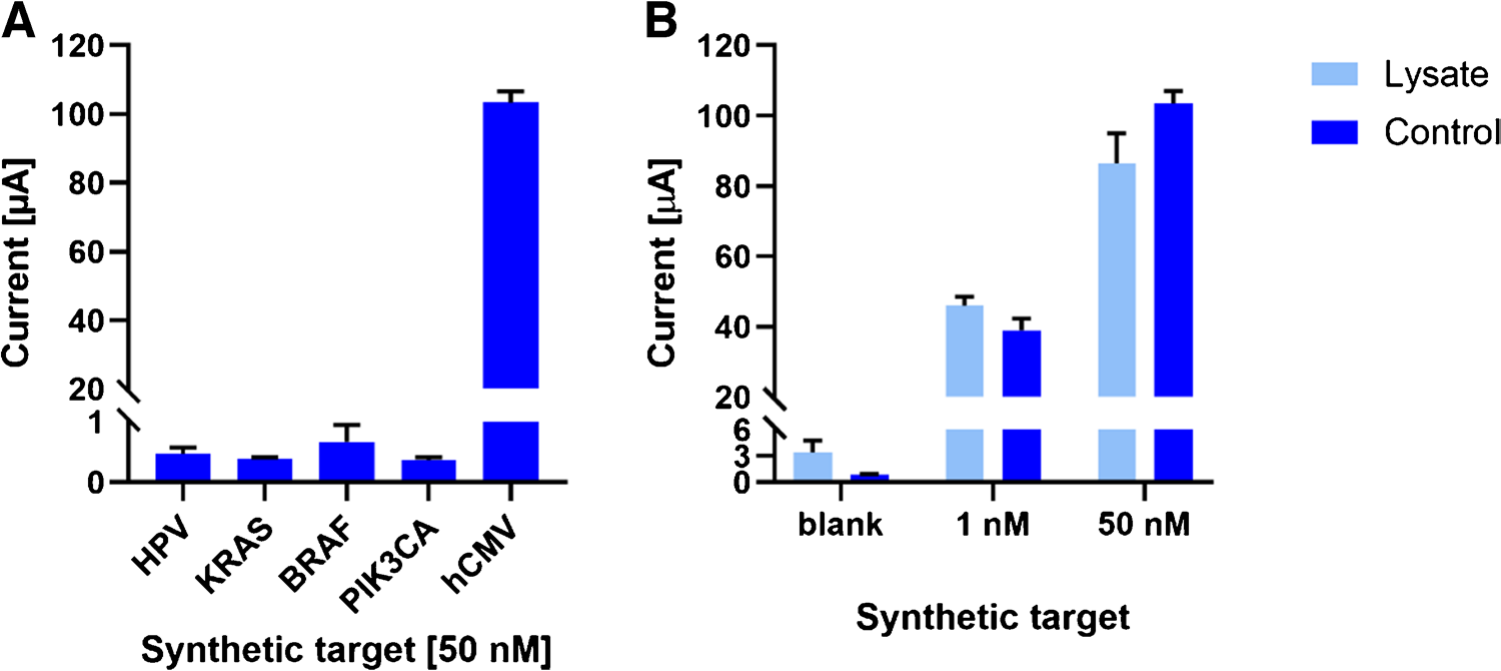
Assay selectivity and robustness of the developed method. **A** Synthetic oligonucleotides corresponding to viral (HPV) and human cancer-associated gene sequences (*KRAS*, *BRAF*, and *PIK3CA*) were incorporated into the hCMV assay to demonstrate the specificity of the capture and detection probes. **B** hCMV synthetic target at concentrations of 1 nM and 50 nM was spiked into the lysate of A375 cell line. The control sample was prepared following the standard protocol without adding the lysate

Figure 5A shows the selectivity of the CP and DP probes toward the hCMV target, showing only negligible binding to noncomplementary human genomic DNA sequences and to the human papillomavirus (HPV) sequence. In addition, to evaluate the robustness of the assay and its ability to minimize false positives in complex biological matrices, we performed a spiking experiment in which two different concentrations of synthetic hCMV target were added to a homogenized cell lysate (Fig. 5B). Compared to the control experiment (standard protocol with no lysate), similar signals were obtained for both target concentrations (118% for 1 nM, 84% for 50 nM). Together, these experiments confirm the high selectivity of the assay and its resistance to nonspecific interactions caused by interfering compounds in complex mixtures.

### Real sample analysis

**Fig. 6:**
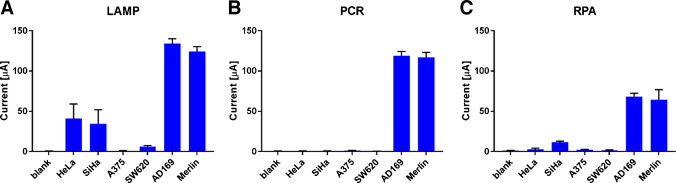
EC measurements. **A** Analysis of LAMP products from cell lines. **B** Analysis of PCR products from cell lines. **C** Analysis of RPA products from cell lines

After establishing the optimal assay conditions for sensitivity, we directly compared three amplification strategies (LAMP, RPA, and PCR) to identify the most effective approach for integration with the biotin-DP system. The optimized approach was tested on a panel of cell lines, including MRC-5 cells infected with hCMV strains AD169 and Merlin (positive controls), two HPV-positive lines (HeLa, HPV18; SiHa, HPV16), and two virus-free lines (A375 and SW620). All samples were independently evaluated for the presence of hCMV by using a commercially available qPCR-based kit (Fig. S7). No amplification was observed in any of the cell lines, except for MRC-5 cells infected with hCMV strains AD169 and Merlin. The linear regression analysis of the kit calibrators demonstrated excellent linearity (R^2^ = 1.0000). Based on the standard curve, the hCMV concentrations were determined to be 1.31 × 10^4^ IU/µL for the AD169 strain and 2.87 × 10^5^ IU/µL for the Merlin strain; however, these observations require extrapolation, as the values lie beyond the validated calibration limits. DNA extracted from the cell lines was introduced into three separate amplification reactions: LAMP, PCR, and RPA, and measured with EC (Fig. 6; see also amperograms in Fig. S8) and ECL readout system (Fig. S9) based on the optimized protocols.

The EC data were in good agreement with the qPCR results; hCMV-infected cell lines produced high current responses that were clearly distinguishable from the negative controls for all amplification strategies—LAMP (Fig. 6A), PCR (Fig. 6B), and RPA (Fig. 6C). Interestingly, HPV-infected cell lines (HeLa and SiHa) also yielded unexpectedly elevated signals in the LAMP assay. Since LAMP employs six primers originally designed for hCMV, partial sequence homology with HPV genomes may have enabled nonspecific amplification. This interpretation is supported by BLAST analysis, which revealed a notable similarity between the LAMP primers and regions of the HPV16 and HPV18 genomes (data not shown). In contrast, nonspecific amplification was not observed with either PCR or RPA. The highest signals were observed for the LAMP products (mean values approx. 130 µA, S/N approx. 167), followed by PCR (approx. 120 µA, S/N approx. 160) and RPA (approx. 65 µA, S/N approx. 49). Owing to its unique amplification mechanism, the LAMP reaction was anticipated to exhibit superior performance, as its complex primer architecture and dumbbell-shaped amplicons generate multiple potential binding sites for DPs on a single strand. The lower RPA signals may be attributed to the shorter amplification time (20 min) compared to LAMP and PCR, as well as the use of shorter F3/B3 primers than is typically recommended; however, the currents remained substantially higher than those of the negative controls.

Another useful metric is the ratio of signal intensity to amplification time (S/T). Although the absolute signal intensities of LAMP and PCR were similar, PCR required approximately triple the time to reach these levels (90 min for PCR vs. 30 min for LAMP). When comparing approximate positive signals, the S/T ratios were 4.33 for LAMP (30 min), 1.33 for PCR (90 min), and 3.25 for RPA (20 min). These values further demonstrate that LAMP provides the most efficient signal generation relative to amplification time. From this perspective, LAMP not only produces higher signals owing to its multi-site amplicon architecture but also achieves superior time-normalized sensitivity.

To minimize variability, EC and ECL measurements were performed on the same day using products from the same amplification reaction. While the EC assay produced clear signals, no measurable ECL response was observed from any amplification reaction (Fig. S9A–C), in contrast to the positive control (50 nM [Ru(bpy)_3_]^2+^; Fig. S9D). To investigate the causes of insufficient ECL signal generation, several experimental conditions were evaluated. Amplified DNA was measured using other electrode materials to evaluate their influence on the bead-based assay, but the same negative outcome was obtained (data not shown).

To exclude the possibility that the [Ru(bpy)_3_]^2+^ label at the 5′ end of the DP interfered with the hybridization of the amplified product, high-resolution melting (HRM) analysis was performed and compared with that of the biotin-DP using a synthetic target (Table S5). All measurements were conducted in triplicate, and the melting temperatures (T_m_) of the DPs used for EC and ECL differed by no more than ± 1.26 °C. Therefore, steric hindrance from the terminal label was ruled out as a potential cause of impaired hybridization.

To optimize the assay and address its limitations, we systematically evaluated different DP concentrations, varying amounts of amplified DNA, and the use of a synthetic target oligonucleotide. The potential quenching effect of carboxylic MBs was first investigated, revealing a significant decrease in the ECL signal upon addition of the MBs to the [Ru(bpy)_3_]^2+^ solution (Fig. S10). Consequently, strategies to release [Ru(bpy)_3_]^2+^-DP after hybridization were tested, including high-temperature denaturation and incubation in deionized water; however, neither approach enabled the successful detection of the labeled amplicons.

Furthermore, immobilization of the [Ru(bpy)_3_]^2+^ label onto the oligonucleotide sequence (DP) may represent an additional limiting factor. Concentration-dependent measurements revealed a substantially higher LOD for the labeled DP (8.88 nM) than for free [Ru(bpy)_3_]^2+^ (0.03 nM, Fig. 2), which likely contributed to the insufficient ECL signals. Previously published articles describing ECL detection of DNA often relied on alternative luminophores [42], ECL-resonance energy transfer (ECL-RET) systems [43], or various nanomaterials [44]. These methods typically employ complex protocol schemes, which are often time consuming and expensive. Similarly, existing publications implementing [Ru(bpy)_3_]^2+^ labeling frequently utilize complex derivatives such as [Ru(phen)_3_]^2+^ for DNA intercalation [45] or [Ru(bpy)_3_]^2+^ doped SiO_2_ nanoparticles [46]. Consequently, the majority of these approaches require complex sample or electrode preparation, often relying on noncommercially available labels. Furthermore, many studies have validated these methodologies using spiked laboratory samples rather than clinical or real samples. The use of [Ru(bpy)_3_]^2+^-labeled DP could enable direct detection of the amplified product and shorten the ECL hybridization protocol; however, costly custom synthesis of DP is required. Similarly, employing other labels with higher emission intensity might enhance sensitivity.

To the best of our knowledge, only one publication [47] has described hCMV detection in clinical samples. This assay was based on PCR amplification followed by ECL measurement of [Ru(bpy)_3_]^2+^-labeled probes in a qPCR cycler using ECL technology. This system was discontinued and replaced with qPCR technology. Other publications focusing on the direct detection of oncoviral DNA have focused on HPV detection [48–51], all of which used additional nanomaterials such as deposited gold nanoparticles (AuNPs), polymer dots, Ru@SiO2 NPs, Au nanocluster probes [50], hybridization chain reaction [49], CRISPR/Cas12a systems for signal activation [48, 51], or spiked samples [49].

Furthermore, the majority of previously reported assays rely on custom-built or in-house instrumentation, rather than commercially available platforms. Crucially, the utility of the ECL instrument used in this study remains underinvestigated in this field. While a study on the general ECL performance of [Ru(bpy)_3_]^2+^ and luminol exists [52], along with articles describing protein detection using luminol-fluorescein or [Ru(bpy)_3_]^2+^-Au@Pt/AuNPs systems [53, 54], none of these studies focus on the detection of nucleic acids or direct labeling using [Ru(bpy)_3_]^2+^.

Although EC detection of hCMV has been described in previous studies, only a few reports have evaluated its performance using real clinical samples. For example, Aztek et al. [33] analyzed clinical blood serum samples (six hCMV-positive and four hCMV-negative) for the presence of hCMV DNA. PCR amplicons were adsorbed onto the SPCEs and hybridized with a biotinylated DNA probe complementary to the target sequence. This complex was subsequently labeled with HRP and the enzymatic reaction was initiated using o-phenylenediamine and H_2_O_2_. The resulting electroactive product, 2,2′-diaminoazobenzene , was quantified by differential pulse voltammetry. This study successfully discriminated between positive and negative clinical samples with an LOD of 3.6 × 10^5^ copies of amplified hCMV DNA/mL. Other studies have relied on in vitro infected cells [55–57] or spiked clinical matrices, such as urine or saliva [58–60]. For example, Pires et al. [59] developed a disposable EC immunosensor for detecting hCMV glycoprotein B in urine samples spiked with recombinant antigens. The assay uses a sandwich-type format on SPCEs with antibody immobilization, gold nanoparticle labeling, and silver-catalyzed anodic stripping voltammetry for signal detection. Unlike previous EC studies, which did not use isothermal amplification, we designed working primers for both isothermal methods and PCR and combined them with a simple and fast EC readout to detect hCMV in infected cancer cell lines. Table S6 further compares our system with other EC-based assays and other analytical techniques in terms of biological material, LOD, and overall assay time.

## Conclusion

In this work, we established a robust, rapid, and user-friendly EC assay capable of detecting hCMV in cancer cell lines, demonstrating its applicability to biologically relevant samples. PCR, LAMP, and RPA amplification strategies were systematically compared, outlining their respective advantages and limitations. By integrating nucleic acid amplification with EC readout, we show that both PCR- and RPA-coupled assays provide high specificity and reliably distinguish hCMV-infected from uninfected cells. Importantly, this study represents the first application of LAMP and RPA in combination with EC detection for hCMV analysis, underscoring the versatility of these amplification strategies within low-instrumentation diagnostic platforms. Although LAMP yielded the highest analytical sensitivity, its nonspecific amplification remains a challenge.

Our findings are consistent with those reported by Aebischer et al. [61], who compared RT-qPCR, LAMP, and RPA for detecting the Schmallenberg virus and bovine viral diarrhea virus. The authors concluded that no single amplification method was universally superior; rather, each technique offered distinct advantages and limitations that must be carefully considered during assay design. Specifically, RT-qPCR demonstrated strong sensitivity and robustness, LAMP combined high sensitivity with low cost and operational simplicity, and RPA provided the fastest reaction times with minimal and portable equipment requirements.

Amperometric detection demonstrated high sensitivity and selectivity. However, ECL analysis did not achieve the required performance under our conditions and requires further optimization. The ECL instrument used in this study has primarily been applied to relatively simple, low-molecular-weight ECL systems, such as luminol-fluorescein assays [53], or monitoring ECL responses based on potassium persulfate or hydrogen peroxide for glutathione detection [62]. To the best of our knowledge, this instrument has not been previously employed for magnetic bead–based DNA ECL assays, which may have contributed to the observed sensitivity limitations. Overall, our results highlight the potential of EC assays, particularly when paired with rapid isothermal amplification, to serve as accessible and sensitive tools for future hCMV screening in tumor-associated samples and point-of-care settings.

## Supplementary Information

Below is the link to the electronic supplementary material.

Supplementary file1 (DOCX 963 KB)

## Data Availability

The authors declare that the data supporting the findings of this study is available within the paper and its Supplementary Information files. Raw data files are available from the corresponding author upon reasonable request.

## Declarations

The cervical cancer cell lines HeLa and SiHa, colorectal adenocarcinoma cell line SW620, human melanoma cell line A375, and lung fibroblast cell line MRC-5 and hCMV laboratory strain AD169 and clinical strain Merlin were all purchased from ATCC, USA.
