# Supplementary material for: Shining a light on viral detection: a comparative study of electrochemical and electrochemiluminescence techniques for human cytomegalovirus
Source: Anal Bioanal Chem. 2026 Mar 5;418(9):2823–37. doi: 10.1007/s00216-026-06410-8 (PMC13079543; doi:10.1007/s00216-026-06410-8)
Supplement: Supplementary file 1 — Supplementary file1 (DOCX 963 KB) [file 216_2026_6410_MOESM1_ESM.docx]

**SUPPLEMENTARY INFORMATION**

**Shining a Light on Viral Detection: A Comparative Study of Electrochemical and Electrochemiluminescence Techniques for Human Cytomegalovirus**

Aneta Fried^+,a,b^, Karolina Itterheimova^+,a,b^, Ludmila Moranova^a^, Martin Bartosik^a,^*^*^*

*^a^ Research Centre for Applied Molecular Oncology, Masaryk Memorial Cancer Institute, Zluty Kopec 7, 656 53 Brno, Czech Republic*

*^b^ National Centre for Biomolecular Research, Faculty of Science, Masaryk University, Kamenice 5, 625 00 Brno, Czech Republic*

** Corresponding author*

^+^authors contributed equally

**Content**

Electrode types

Cyclic voltammetry

Table S1: List of used oligonucleotide sequences

Table S2: List of optimized conditions with selected optimal parameter

Table S3: Reproducibility of LAMP reaction with EC readout

Table S4: EC signals of synthetic target

Table S5: High resolution melting analysis of detection probes with synthetic target

Table S6: Comparison of hCMV DNA detection assays

Figure S1: ECL scan rate

Figure S2: Buffer composition for ECL measurement

Figure S3: Comparison of various materials of screen printed electrodes

Figure S4: ECL measurements of [Ru(bpy)_3_]^2+^ concentration dependence measured on spectrophotometer

Figure S5: Raw signals of ECL detection of 50 nM [Ru(bpy)_3_]^2+^ with ECL and EC readout

Figure S6: Cyclic voltammetry of ECL measurements

Figure S7: Analysis of hCMV strains using a commercially available qPCR kit

Figure S8: A: Raw amperometric signals obtained by assays involving LAMP, PCR, and RPA

Figure S9: ECL measurements of amplified products and positive control

Figure S10: Quenching effect of MBs

References

*Electrode types*

An additional set of modified screen printed electrodes (besides DRP-110) included DRP-110CNF, DRP-110GNP, DRP-110CNF-GNP, DRP-110GPH, DRP-220AT and DRP-550 (all from Metrohm DropSens).

*Cyclic voltammetry*

Cyclic voltammetry was performed in the potential range from +0.3 V to +1.8 V using a scan rate of 0.5 V/s and E_step_ of 0.002 V.

**Table S1:** List of oligonucleotide sequences used in the study.

| **Name** | **Sequence 5´- 3´** | **5’ modification** |
| --- | --- | --- |
| hCMV_US28_F3 | TGTTTCTGTACGGCGTTGTC |  |
| hCMV_US28_B3 | TGGCTAGGGAGTTGTGATCT |  |
| hCMV_US28_FIP | ATCCGACGTCGCCAGGTGATTTTCTCTTCGGTTCCATCGG |  |
| hCMV_US28_BIP | TACTTTATCAACCTCGCGGCCGTGCATCCACAGAGGTAGTGT |  |
| hCMV_US28_LF | GTGAAGATCACCAAGAAGTT |  |
| hCMV_US28_LB | CGATTTGCTTTTCGTTT |  |
| hCMV_US28_NH2-CP | AACATCGCCGGAGCATTG | aminoC6sp |
| Biotin-DP | GTGAAGATCACCAAGAAGTT | biotin |
| [Ru(bpy)_3_]^2+^-DP | GTGAAGATCACCAAGAAGTT | Tris(bipyridine)ruthenium(II) chloride |
| hCMV_oligo_target | TTCCATCGGCAACTTCTTGGTGATCTTCACCATCACCTGGCGACGTCGGATTCAATGCTCCGGCGATGTTTACTTTATCA |  |
| hCMV_oligo_complement | TGATAAAGTAAACATCGCCGGAGCATTGAATCCGACGTCGCCAGGTGATGGTGAAGATCACCAAGAAGTTGCCGATGGAA |  |
| DP1 | GATCTAGGAGGTATTGCAT | biotin |
| DP2 | ATGCAATACCTCCTAGATC | biotin |
| DP3 | CTTTTCGTTTGTACACTACC | biotin |
| hCMV_US28_RPA_F | TTAATCAGTCAAAGCCAGTTACGTTGTTTC |  |
| hCMV_US28_RPA_R | AGAGGTAGTGTACAAACGAAAAGCAAATC |  |
| HPV_target | ATAATGAGCTGACAGATGAAAGCGATATGGCATTTGAATATGCCTTATTAGCAGACAGCAACAGCAATGCAGCTGCCTTTTTAAAAAGCAATTGCCAAGCTAAATATTTAAAAGATTGTGCCACAATGTGCAAACATTAT |  |
| KRAS_target | TGGAGCTAGTGGCGTAGGCAAGAGTGCCTTGACGATACAGCT |  |
| BRAF_target | CTTTACTTACTACACCTCAGATATATTTCTTCATGAAGACCTCACAGTAAAAATAGGTGATTTTGGTCTAGCTACAGTGAAATCTCGATGGAGTGGG |  |
| PIK3CA_target | GACAATGAATTAAGGGAAAATGACAAAGAACAGCTCAAAGCAATTTCTACACGAGATCCTCTCTCTGAAATCACTGAGCAGGAGAAAGATTT |  |

**Table S2:** List of optimized parameters.

| **Parameter** | **Tested condition** | **Selected condition** |
| --- | --- | --- |
| **Amplification reaction** | | |
| Loop primers | With; Without | With loop primers |
| LAMP incubation temperature (°C) | 62; 64 | 62 |
| **Hybridization step** | | |
| DP concentration (µM) | 0.05; 0.1; 0.5; 1; 5 | 0.5 |
| Incubation with DP | One-step; Two-step | One-step |
| Hybridization temperature (°C) | RT; 30; 40; 50 | 40 |
| Target hybridization time (min) | 15; 30; 60 | 15 |
| NaCl concentration for target hybridization (M) | 0.3; 0.6 | 0.6 |
| MB surface blocking^a^ | Scheme A; Scheme B | Scheme B |
| DP sequence | Biotin/[Ru(bpy)_3_]^2+^-DP; DP1; DP2; DP3 | Biotin/[Ru(bpy)_3_]^2+^-DP |
| Target denaturation | 95 °C (10 min); none | 95 °C (10 min) |
| **EC measurement** | | |
| Biotin-label format | dUTP; DP | DP |
| STR-HRP conjugate | STR-HRP (monomer); SPP-T; SPP-S | SPP-T |
| SPP dilution | 1:250; 1:500; 1:1000; 1:2000 | 1:1000 |
| **ECL readout** | | |
| Scan rate (V/s) | 0.05; 0.1; 0.25; 0.5 | 0.5 |
| ECL buffer | 80 µM TPA; 40 mM TPA; 40 mM TPA + 0.1 % Tween 20 | 40 mM TPA |
| Electrode type | C; CNF-C; AuNP-C; AuNPs-CNF-C; GPH-C; Au; Pt | C |
| Measurement setup | Spectrophotometer;  Photodiode 1×; 10×; 100× amplification | Photodiode 100× amplification |

^a^ Scheme A: 3× 100 µL WB + 3× 100 µL CBB; Scheme B: 2× 100 µL WB, 2× 100 µL CBB + 3× 100 µL CBB

**Table S3:** Reproducibility of LAMP reaction with EC readout.

|  | **Current, μA** | | | | **Mean, μA** | **SD, μA** | **RSD, %** | **S/N** |
| --- | --- | --- | --- | --- | --- | --- | --- | --- |
| **blank** | 1.469 | 0.931 | 1.489 | 0.881 | 0.927 | 0.488 | 52.553 | 137.99 |
|  | 1.532 | 0.429 | 0.337 | 0.225 |  |  |  |  |
|  | 1.571 | 1.059 | 0.874 | 0.335 |  |  |  |  |
| **AD169** | 119.088 | 126.875 | 136.875 | 135.35 | 127.913 | 7.469 | 5.839 |  |
|  | 122.613 | 125.968 | 127.8 | 111.75 |  |  |  |  |
|  | 127.612 | 139.375 | 133.4 | 128.25 |  |  |  |  |

**Table S4:** EC signals of synthetic target.

| **Concentration**  **of synthetic target, nM** | **Current, μA** |
| --- | --- |
| 0 (blank) | 0.834 ± 0.145 |
| 0.1 | 4.688 ± 0.632 |
| 0.25 | 8.888 ± 0.405 |
| 0.5 | 26.158 ± 1.799 |
| 1 | 38.996 ± 3.304 |
| 2.5 | 56.854 ± 3.124 |
| 5 | 66.355 ± 7.669 |
| 10 | 89.175 ± 3.990 |
| 25 | 101.850 ± 0.639 |
| 50 | 119.604 ± 13.280 |
| 100 | 113.483 ± 2.559 |
| 250 | 107.169 ± 10.507 |
| 500 | 125.354 ± 5.694 |

**Table S5:** HRM analysis of detection probes with synthetic target.

|  | **Mean T_m_, °C** | **SD, °C** | **RSD, %** | **ΔT_m_, °C** |
| --- | --- | --- | --- | --- |
| **[Ru(bpy)_3_]^2+^-DP** | 62.83 | 0.17 | 0.3 | 1.26 |
| **Biotin-DP** | 64.09 | 0.07 | 0.1 |  |

**Table S6:** Comparison of hCMV DNA detection assays.

| **Method** | **Principle** | **Material type** | **LOD** | **Assay time** | **Reference** |
| --- | --- | --- | --- | --- | --- |
| EC | PCR; hybridization on microwell plate; label - alkaline phosphatase | MRC5 cells infected by AD169 | 10 amol/mL | PCR (~60 min) + 110 min | [1] |
| EC | PCR; hybridization with oligonucleotide-modified Au probe; release of Au ions | Cell culture (not specified) | 5 pM | PCR (N/A) + 45 min | [2] |
| EC | PCR; adsorption of amplified DNA  biotin-probe; Streptavidin-HRP | MRC5 cells infected by AD169 | 0.6 amol/mL | Overnight + PCR (~60 min) + 90 min | [3] |
| ECL | [Ru(bpy)_3_]^2+^-labeled probes; streptavidin MBs | Serum, plasma, cerebrospinal fluid, whole blood  cell culture | 25 viral particles/mL | PCR (~80 min) + 60 min | [4] |
| qPCR | GeneProof Cytomegalovirus (CMV) PCR Kit | Bronchoalveolar lavage fluid | 100 copies/mL  79.4 IU/mL | PCR (~90 min) | [5] |
| Multiplex PCR | PCR; fluorescence readout | Cerebrospinal fluid | 340 viral particles/mL | PCR (~45 min) | [6] |
| Lateral flow strip | Multienzyme isothermal rapid amplification | Breast milk | 5 × 10^3^ copies/µL | 22 min | [7] |
| Multiplex real-time PCR | PCR; fluorescence readout | Blood | 100 copies/µL | PCR (~35 min) | [8] |
| EC | Isothermal amplification; biotin-probe; streptavidin-HRP | MRC5 cells infected by AD169/Merlin | 10 pM | RPA (20 min) + 41 min | This work |


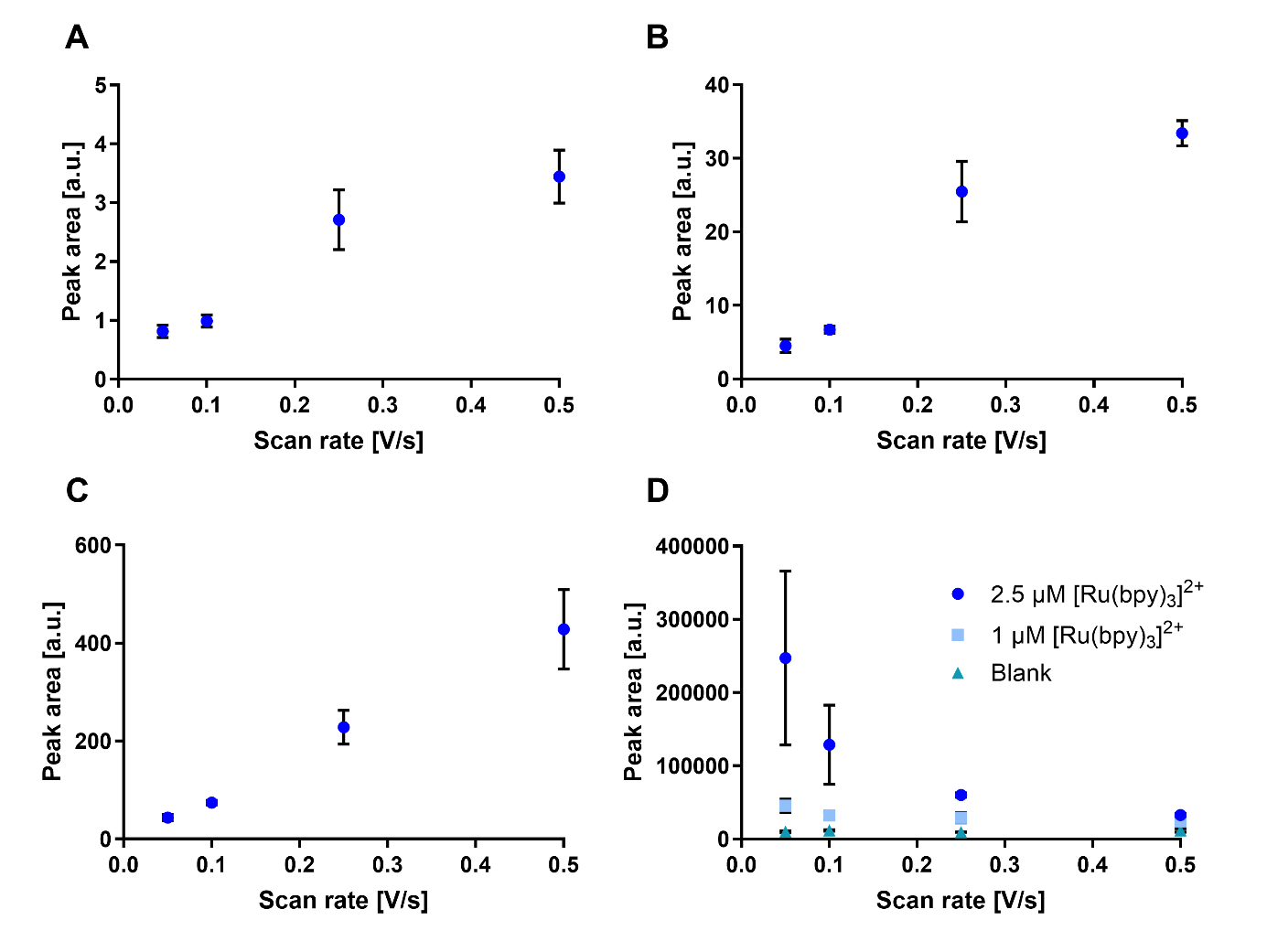


**Figure S1**: ECL scan rate optimization. A: Photodiode without signal amplification (1×). B: Photodiode with 10× signal amplification. C: Photodiode with 100× signal amplification. D: Spectrophotometer. Concentration of TPA was 40 mM.


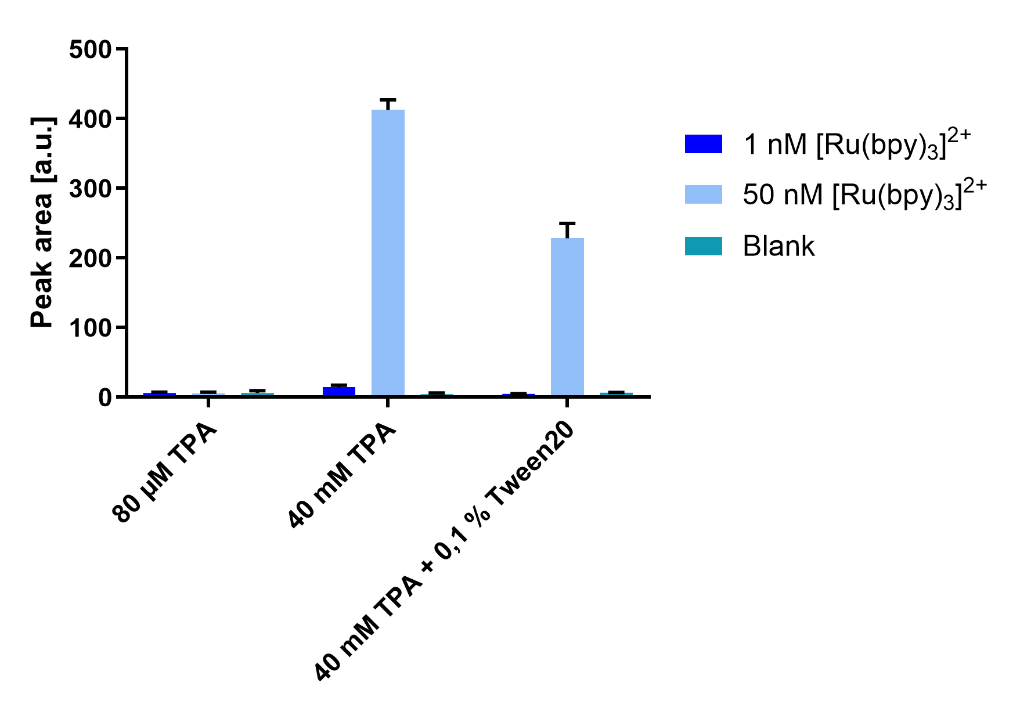


**Figure S2**: Buffer composition for ECL measurement based on literature recommendation.


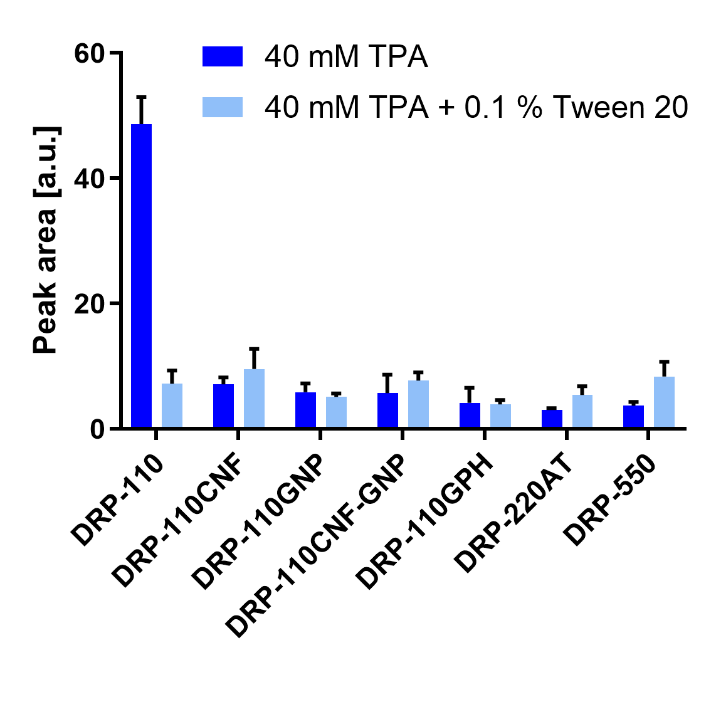


**Figure S3**: Comparison of various materials of screen printed electrodes using 10 nM [Ru(bpy)_3_]^2+^. 110 – unmodified carbon; 110CNF – carbon nanofibers-modified carbon; 110GNP – gold nanoparticles-modified carbon; 110CNF-GNP – carbon nanofibers and gold nanoparticles-modified carbon; 110GPH – graphene-modified carbon; 220AT – gold; 550 – platinum.


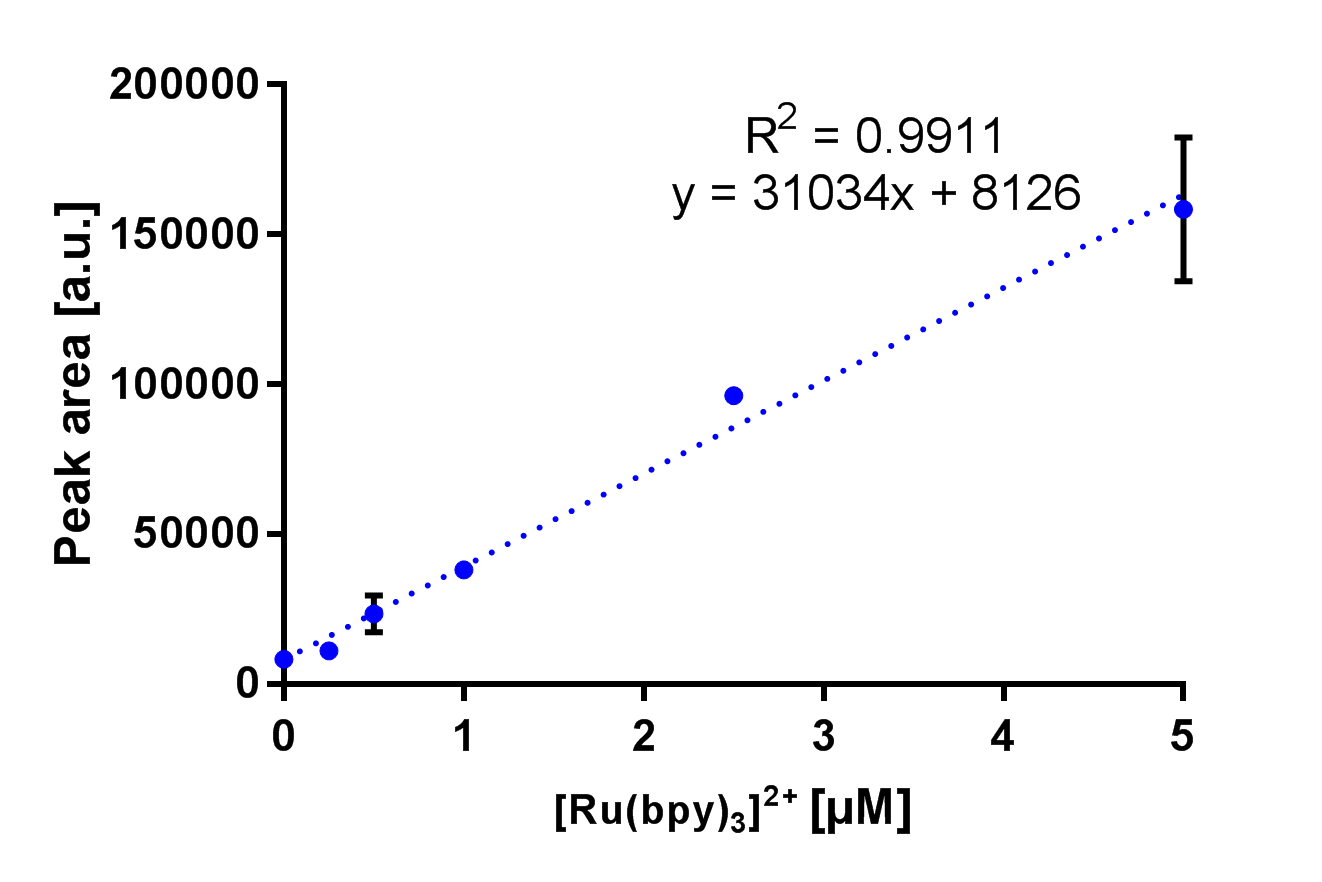


**Figure S4:** ECL measurements of [Ru(bpy)_3_]^2+^ concentration dependence. Measured on spectrometer in the range of 0.25-5 µM. Concentration of TPA was 40 mM.


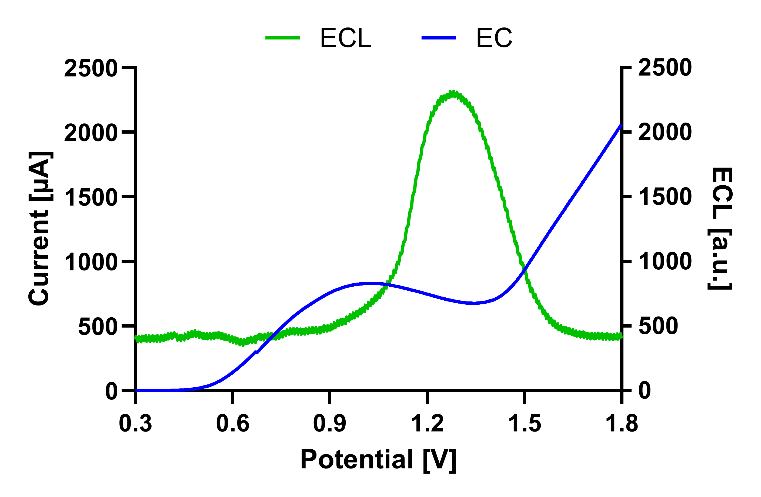


**Figure S5**: Raw signals of 50 nM [Ru(bpy)_3_]^2+^/40 mM TPA coupled with ECL (green) and EC (blue) readouts.


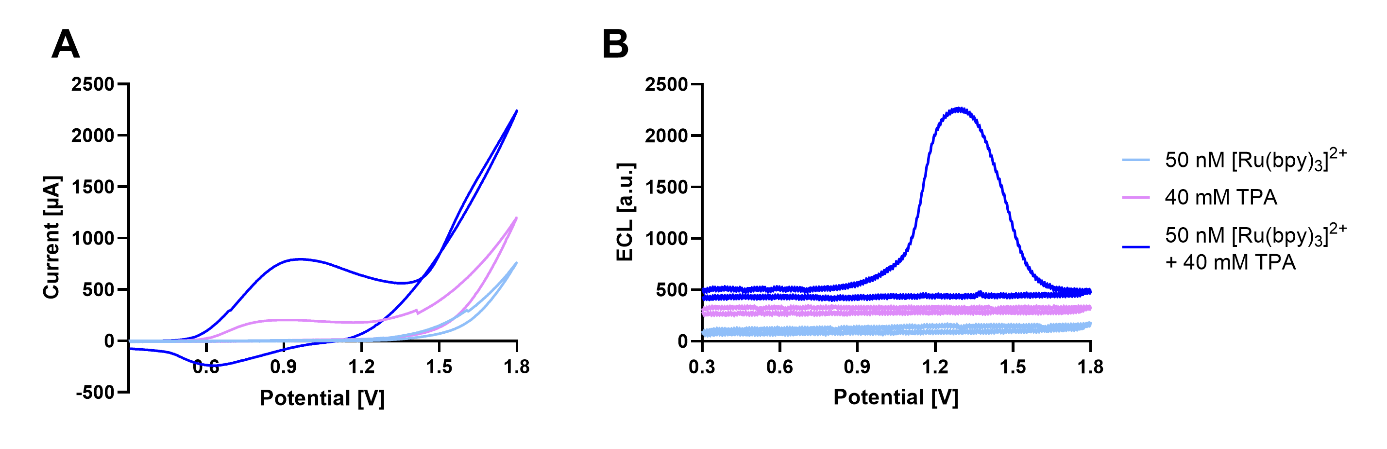


**Figure S6**: Electrochemical (A) and electrochemiluminescence (B) signals of cyclic voltammetry of 50 nM [Ru(bpy)_3_]^2+^ alone, 40 mM TPA alone, and a mixture of 50 nM [Ru(bpy)_3_]^2+^ and 40 mM TPA.

**
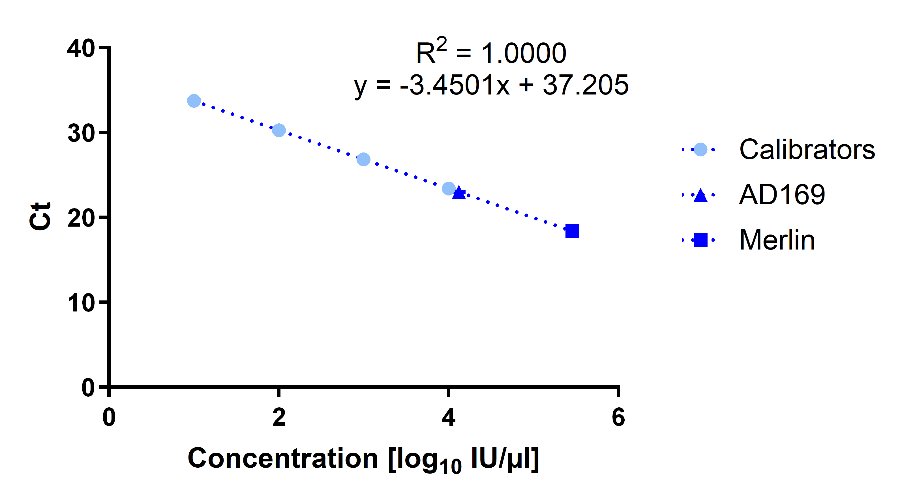
**

**Figure S7:** Linear regression analysis of hCMV strains AD169 and Merlin using a commercially available qPCR kit (GeneProof, IVDR certified). No amplification of negative samples was observed. Each value represents the mean of technical triplicates. The corresponding standard deviations are extremely small and thus not visible at the scale of the plot. Concentration of TPA was 40 mM.


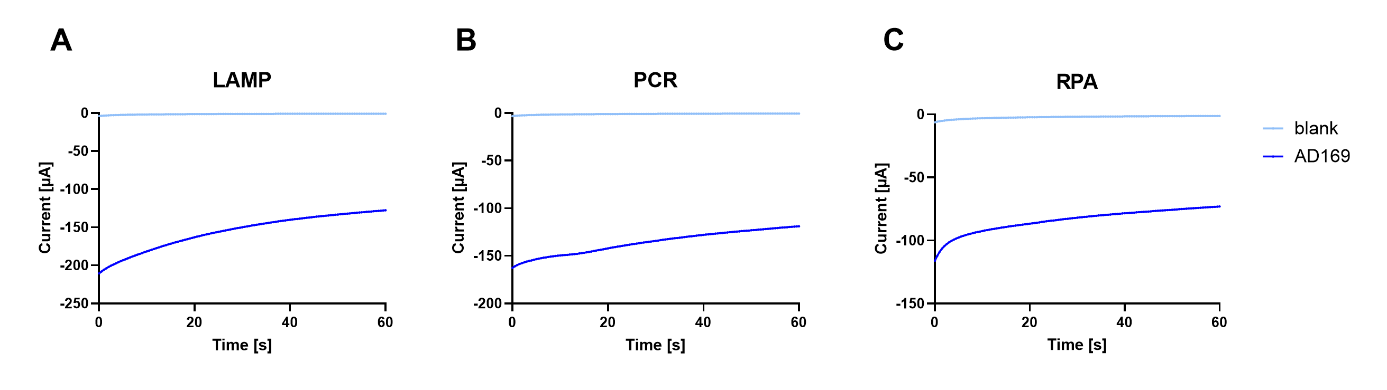


**Figure S8**: Raw amperometric signals obtained by assays involving (A) LAMP, (B) PCR, and (C) RPA. As an input, 100 ng of DNA extracted from MRC-5 cell line infected with AD169 was used.


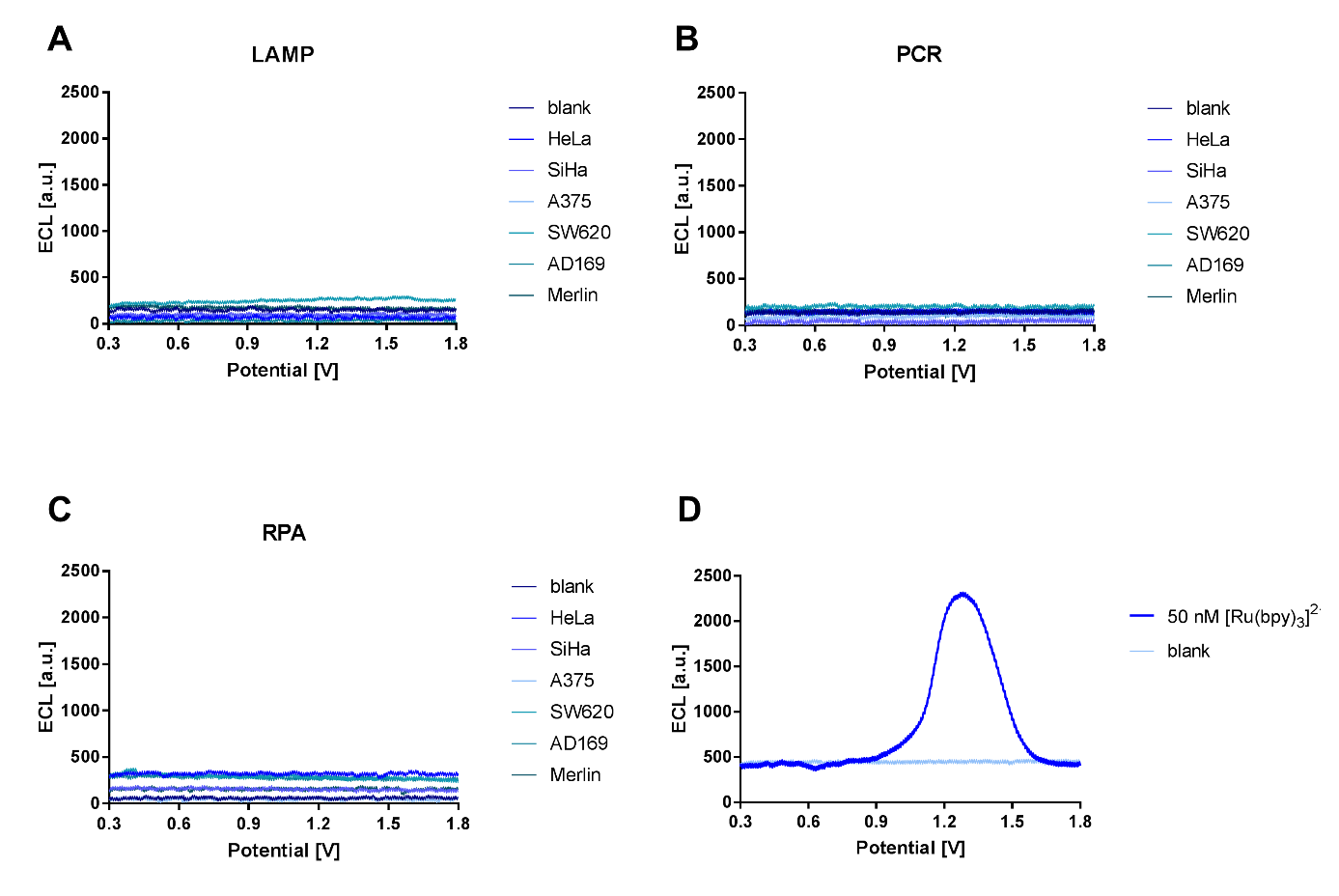


**Figure S9:** ECL measurements. A: Analysis of LAMP products from cell lines. B: Analysis of PCR products from cell lines. C: Analysis of RPA products from cell lines. D: ECL curves for 50 nM [Ru(bpy)_3_]^2+^ and blank on photodiode with 100× amplification. Concentration of TPA was 40 mM.


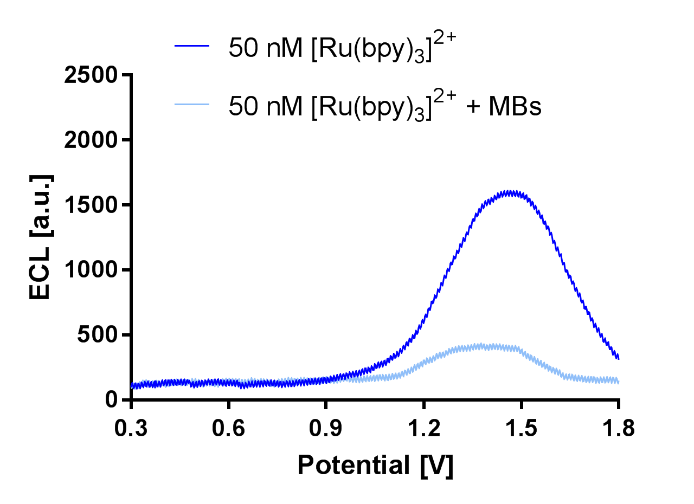


**Figure S10**: Quenching effect of MBs. Concentration of TPA was 40 mM.

**References**

1. Bagel O, Degrand C, Limoges B, Joannes M, Azek F, Brossier P (2000) Enzyme Affinity Assays Involving a Single-Use Electrochemical Sensor. Applications to the Enzyme Immunoassay of Human Chorionic Gonadotropin Hormone and Nucleic Acid Hybridization of Human Cytomegalovirus DNA. Electroanalysis 12 (18):1447-1452. <https://doi.org/10.1002/1521-4109(200012)12:18><1447::AID-ELAN1447>3.0.CO;2-D

2. Authier L, Grossiord C, Brossier P, Limoges B (2001) Gold Nanoparticle-Based Quantitative Electrochemical Detection of Amplified Human Cytomegalovirus DNA Using Disposable Microband Electrodes. Anal Chem 73 (18):4450-4456. <https://doi.org/10.1021/ac0103221>

3. Azek F, Grossiord C, Joannes M, Limoges B, Brossier P (2000) Hybridization Assay at a Disposable Electrochemical Biosensor for the Attomole Detection of Amplified Human Cytomegalovirus DNA. Anal Biochem 284 (1):107-113. <https://doi.org/10.1006/abio.2000.4692>

4. Boom R, Sol C, Weel J, Gerrits Y, Boer Md, Dillen PW-v (1999) A Highly Sensitive Assay for Detection and Quantitation of Human Cytomegalovirus DNA in Serum and Plasma by PCR and Electrochemiluminescence. J Clin Microbiol 37 (5):1489-1497. <https://doi.org/10.1128/jcm.37.5.1489-1497.1999>

5. Zak P, Vejrazkova E, Zavrelova A, Pliskova L, Ryskova L, Hubacek P, Stepanova V, Kostal M, Koblizek V, Paterova P, Radocha J (2020) BAL fluid analysis in the identification of infectious agents in patients with hematological malignancies and pulmonary infiltrates. Folia Microbiol 65 (1):109-120. <https://doi.org/10.1007/s12223-019-00712-4>

6. Luzius T, Jeske SD, Baer J, Goelnitz U, Protzer U, Wettengel JM (2025) A Multiplex Polymerase Chain Reaction Assay for the Detection of Herpes Simplex Virus, Cytomegalovirus, and Varicella-Zoster Virus in Cerebrospinal Fluid. Microorganisms 13 (1):111. <https://doi.org/10.3390/microorganisms13010111>

7. Liu M-h, Guo X, Sun M-l, Li J-l, Liu S-h, Chen Y-z, Wang D-y, Wang L, Li Y-z (2024) Rapid detection of human cytomegalovirus by multienzyme isothermal rapid amplification and lateral flow dipsticks. Front Cell Infect Microbiol Volume 14 - 2024. <https://doi.org/10.3389/fcimb.2024.1430302>

8. Xu Y, Lv Y, Lin M, Li M, Cui D, Wang Y, Shen C, Xie J (2024) A novel multiplex real-time PCR assay for the detection of cytomegalovirus, Epstein-Barr virus, herpes simplex virus 1/2 and strategies for application to blood screening. Diagn Microbiol Infect Dis 109 (1):116234. <https://doi.org/10.1016/j.diagmicrobio.2024.116234>
